# Medicinal Plants as a Potential and Successful Treatment Option in the Context of Atherosclerosis

**DOI:** 10.3389/fphar.2020.00403

**Published:** 2020-04-08

**Authors:** Tatiana V. Kirichenko, Vasily N. Sukhorukov, Alexander M. Markin, Nikita G. Nikiforov, Ping-Yen Liu, Igor A. Sobenin, Vadim V. Tarasov, Alexander N. Orekhov, Gjumrakch Aliev

**Affiliations:** ^1^ Laboratory of Infection Pathology and Molecular Microecology, Research Institute of Human Morphology, Moscow, Russia; ^2^ Laboratory of Angiopathology, Institute of General Pathology and Pathophysiology, Moscow, Russia; ^3^ Laboratory of Medical Genetics, National Medical Research Center of Cardiology, Moscow, Russia; ^4^ Institute of Clinical Medicine, College of Medicine, National Cheng Kung University, Tainan, Taiwan; ^5^ Division of Cardiology, College of Medicine, National Cheng Kung University Hospital, Tainan, Taiwan; ^6^ Sechenov First Moscow State Medical University (Sechenov University), Moscow, Russia; ^7^ Institute of Physiologically Active Compounds, Russian Academy of Sciences, Chernogolovka, Russia; ^8^ GALLY International Research Institute, San Antonio, TX, United States; ^9^ Laboratory of Molecular Pathology, Research Institute of Human Morphology, Moscow, Russia

**Keywords:** medicinal plants, atherosclerosis, cardiovascular risk, anti-atherosclerotic mechanisms, carotid IMT

## Abstract

Atherosclerosis is a chronic multifactorial disease characterized by mainly changes of blood lipids profile and inflammation in vessel wall. The cardiovascular disease based on atherosclerosis is currently the leading cause of mortality in developed countries. Therefore, timely prevention and therapy of atherosclerosis are able to reduce the risk of the development of its clinical manifestations. Anti-atherosclerotic activity of medicinal plants mainly appears in their multiple effects such as anti-inflammatory, antioxidant, anti-atherogenic, hypotensive, lipid-lowering, anti-thrombotic. Moreover, most of medicinal plants are characterized by their pleiotropic anti-atherosclerotic action. In addition, the medicinal plants-derived pharmacological substances and/or compounds are characterized by relative safety and fewer side effects that allows considering them as one of potential anti-atherosclerotic effective agents. The direct anti-atherosclerotic effect of some medicinal plants was confirmed in clinical trials of carotid Intima-media thickness (IMT) progression during long-term medication with medicinal plants. This review attempted to determine the current status of the databases PubMed and Scopus (until November, 2019) to investigate the medicinal plants possessing anti-atherosclerotic activity in experimental and clinical studies.

## Introduction

Atherosclerosis is a chronic multifactorial disease characterized by dyslipidemia and inflammation. Nowadays it is one of the key reasons of cardiovascular disease that is currently the leading cause of mortality in developed countries (Libby et al., 2011; Fredman and Spite, 2013). Atherosclerosis develops in the human arteries for many years, and remains asymptomatic for a long time. Patients very often learn about the presence of atherosclerotic lesions when they seek medical attention because of the development of cardiovascular disease, that is a complication of the atherosclerotic process. However, initial atherosclerotic lesions can be identified at the subclinical stage by modern diagnostic methods, which make it possible to start prevention timely. Anti-atherosclerotic therapy is very important for the prevention of diseases of this group and their complications. Non-drug methods of prevention exist and apply. They are based on the prevention of the development of cardiovascular disease by changing lifestyle, keeping a diet, and administration of courses of medicinal plants and natural products that possess anti-atherosclerotic properties (Pogosova et al., 2017).

In general, atherosclerosis can be also described as a type of immunological disorder, in which immune cells are activated along the inflammatory pathway and migrate into the vascular intima. After that, atherosclerotic lesion may develop in this place (Frostegard, 2013). Recent data indicate that the prevailing beliefs about the decisive role of high cholesterol in the development of atherosclerosis are not entirely correct. High blood cholesterol does not always clearly correlate with the presence of atherosclerotic lesions in arterial wall and complications of atherosclerosis process (Orekhov and Ivanova, 2017).

Currently, a strategy for the correction of lipid metabolism disorders is widely used around the world to treat atherosclerosis. But at the same time, researchers are developing methods to counteract inflammatory processes in the vessel wall at cellular level. The viability of these methods is indicated by the latest data from studies of the pathogenesis of atherosclerotic lesions. This process begins in the intima layer of large and medium arteries, especially in places of bifurcation. Vascular wall of such areas experiences increased stress due to the turbulent effects of blood flow. This causes damage to the cells of endothelial layer of intima, resulting in attracting of immune cells due to the production of adhesion molecules. (Koch and Zernecke, 2014). At the same time, low-density lipoproteins (LDL) undergo modification (Nikifirov et al., 2017; Alipov et al., 2017) and acquire the ability to penetrate and accumulate at the subendothelial level of the arterial wall. There they can additionally undergo oxidation, turning into oxidized LDL. Monocytes attracted by endothelial cells in the intimal space differentiate into macrophages and absorb oxidized LDL. However, in the event of a violation of the processes of cholesterol metabolism in macrophages, they are transformed into foam cells (Pirillo et al., 2013; Lopes-Virella and Virella, 2013; Witztum and Lichtman, 2014; Akhter et al., 2016; Tabas and Lichtman, 2017).

Thus, the activation of cascades of immune and inflammatory reactions is crucial for the progression of atherosclerotic lesions. In attempt to influence these processes, several new strategies for the treatment of atherosclerosis, based on the use of various therapeutic agents to inhibit the pro-inflammatory activation of immune cells or stimulate signals to the anti-inflammatory activity of body systems, have recently appeared. They can be used as an addition to conventional methods of treating cardiovascular diseases based on the use of lipid-lowering preparations (Li et al., 2017; Chistiakov et al., 2018). However, the most common preparations used for anti-atherosclerotic therapy are currently statins since they possess multiple anti-atherosclerotic properties (Davignon, 2012; Tian et al., 2012; Oesterle et al., 2017), but their prescription is still limited because of prolonged administration of preparations of this group leads to a risk of undesirable side effects so statins are traditionally prescribed to patients with hypercholesterolemia (Stroes et al., 2015; Beckett et al., 2015).

Nowadays, there are no preparations that can be used for long-term prevention and treatment of atherosclerosis without the risk of developing pronounced side effects. This leads to the extremely high relevance of the study and use of medicinal plants for long-term anti-atherosclerotic therapy. Actually, the medical properties of various plants have been known since ancient times. A large number of herbal preparations are capable of exerting inhibitory effects in pathological immune reactions, and some of them have traditionally been used for the modulation of atherosclerosis progression. It can be explained by their relative safety, fewer side effects, and, in a sense, their greater effectiveness. Since cytokines are the main mediators in all types of inflammation, that is a significant factor at all phases of the atherosclerosis progression, the studies of the use of medicinal plants for modulation of cytokines production are very relevant in the field of atherosclerosis medication (Ramji and Davies, 2015; Fatkhullina et al., 2016). In addition, medicinal plants are widely used for the correction of conventional risk factors of atherosclerosis such as hyperlipidemia, hypertension, and etc. (Tariq et al., 2016; Chukwuma et al., 2019). The variety of clinical trials' results of various research groups studying medicinal plants can be partially explained by the multiple mechanisms of action on various cardiovascular risk factors. In addition, some visualization methods give encouraging results. For example, evaluation of the carotid intima-media thickness (CIMT) indicator (intima-media thickness of the carotid arteries) using ultrasound investigation can quantify the progression of atherosclerotic lesions and evaluate the direct effect of these preparations on atherosclerosis (Bots et al., 2016; Katakami et al., 2019).

This review reflects current views on how medicinal plants are used to reduce the risk of atherosclerosis development, their direct or indirect anti-atherosclerotic effects. English scientific literature was searched using electronic databases PubMed and Scopus by keywords “medicinal plants”, “natural products”, “atherosclerosis”, “intima-media thickness”, “cardiovascular risk”, “clinical trial” in different combinations until November, 2019, with first focus on medicinal plants with proven direct anti-atherosclerotic activity in patients with carotid atherosclerosis and natural products possessing cardioprotective effects in clinical trials, and further their anti-atherosclerotic mechanisms of action were reviewed. We will focus on medicinal plants and the products created on their base, used in modern practice meeting the principles of evidence-based medicine.

## Possible Anti-atherosclerotic Mechanisms of Medicinal Plants

**Figure 1:**
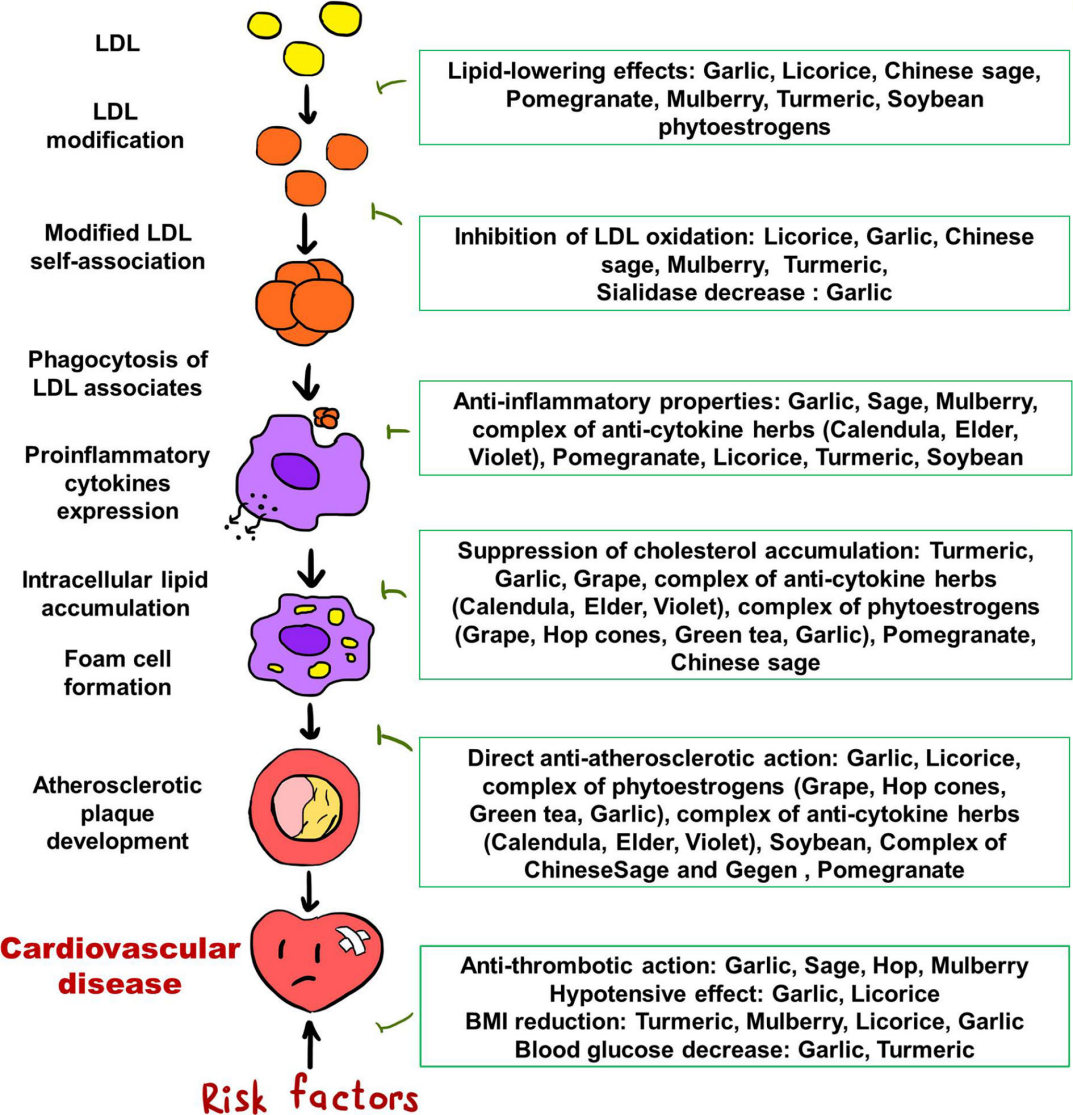
Plausible effects of medicinal plants on different stages of atherosclerosis development.

Atherosclerosis is a multifactorial process, and despite the great progress in understanding the mechanisms of the development of atherosclerotic lesions in the last decades, the pathogenesis of atherosclerosis is the subject of study by a large circle of scientists around the world. Nevertheless, the key points of the pathogenesis of atherosclerosis are known, and the possible pathogenetic mechanisms of the anti-atherosclerotic effects of medicinal plants are being studied in cell cultures, animal models, and human studies. **Figure 1** summarizes plausible effects of medicinal plants reviewed in this article, on different stages of atherosclerosis development. The effects of the most promising natural anti-atherosclerotic agents are noteworthy.

Numerous studies describe the contribution of pro-inflammatory cytokines to the formation of atherosclerotic lesions in arterial wall. Evidence from these studies underlies the potential use of anti-cytokine therapy that can become a promising direction for the correction of changes at early stages of the atherosclerosis development. Thanks to recent studies, it was found that components of a large number of medicinal plants are capable of modulating the pathways of the inflammatory response. Many of these natural products have anti-cytokine mechanism of action, and most of them do not possess pronounced side effects and can be used for long-term prevention and treatment of atherosclerosis. In particular, herbal preparation Inflaminat, mixture of black elder (*Sambucus nigra* L.), violet tricolor (*Viola tricolor* L.), and calendula (*Calendula officinalis* L.), significantly inhibits the expression of inflammatory cytokines *via* inhibiting IL-6 and TNF-α expression and alleviates serum atherogenic activities *via* unknown mechanism in *ex vivo* model (Kirichenko et al., 2016).

There are some beneficial data of multiple anti-inflammatory and anti-atherosclerotic effects of glabridin, a flavonoid isolated from licorice (*Glycyrrhiza glabra* L.). Glabridin possesses anti-inflammatory action by preventing TNF-α-stimulated gene expression of VCAM-1 (vascular cell adhesion molecule-1) and ICAM-1 (intercellular adhesion molecule-1) *via* blocking JNK and NF-κB (Kwon et al., 2007; Cook-Mills et al., 2011). Glabridin possesses anti-cytokine activity, since it suppresses the LPS-stimulated secretion of inflammatory cytokines TNF-α and IL-1β in microglial cells (Park et al., 2010) and TNF-α- stimulated production of adhesion molecules in human umbilical vein endothelial cells (Jong et al., 2006; Chang et al., 2010). In addition to anti-inflammatory properties, glabridin can inhibits LDL oxidation by suppression of 2,2-azobis(2-amidinopropane) hydrochloride (AAPH)--stimulated production of cholesteryl linoleate hydroperoxide in LDL, and the reduction of LDL oxidation by glabridin was confirmed in *ex vivo* model: the ability to oxidize LDL in cell culture of mice peritoneal macrophages decreased after accumulation of glabridin (Rosenblat et al., 1999; Kang et al., 2015).

Garlic (Allium sativum L.) possesses multiple anti-atherosclerotic effects. Allicor, garlic powder, inhibits inflammation signaling (like TNF, IL-1β, ICAM-1, and HLA-DR expression and secretion) (Karagodin et al., 2016a). Garlic also exhibits hypotensive activity through the inhibition of angiotensin-converting enzyme (Asdaq and Inamdar, 2010), positive activation of the growth suppressor p27, descent of ERK1/2 phosphorylation (Castro et al., 2010), and down-regulation of angiotensin II receptor (Mansour et al., 2013), reduction of vasoconstrictor prostanoids synthesis (Al-Qattan et al., 2001), stimulation of hydrogen sulfide (H2S) production (Al-Qattan et al., 2006), and regulation of endothelial nitric oxide (NO) synthesis (Ried and Fakler, 2014; Karagodin et al., 2016a). Garlic antithrombotic activity revealed through suppression of cyclooxygenase (Ali et al., 2000), diminished production of thromboxane B2 and vasoconstrictors like prostaglandin E2 and leukotriene C4 by platelets (Vilahur and Badimon, 2013), control of membrane phospholipases activity, and serotonin and coagulation factor IV secreting from platelets (Rahman, 2007). Aged garlic extract also prevented platelet aggregation by increasing cyclic nucleotides (Allison et al., 2012; Rahman et al., 2016), the quantity of both extracellular ATP and intra- and extracellular thromboxane B2, by inhibiting GPIIb/IIIa receptor and fibrinogen binding, and by abolishing phosphorylation of collagen-induced ERK, JNK, and p38 (Morihara and Hino, 2017). Moreover, antioxidant activity, synthesis of connective tissue matrix components, and cellular proliferative activity are suppressed, and LDL becomes less prone to oxidation thanks to the anti-atherosclerotic activity of garlic extracts (Orekhov et al., 2013; Orekhov et al., 2015). Garlic powder preparations inhibited neointimal thickening in cholesterol-fed rabbit models (Sobenin et al., 2016a). Garlic powder obviated neointimal thickening in cholesterol-fed rabbits, and Allicor (garlic tablet) decreased the accumulation of cholesterol in cell cultures incubated with serum from patients with atherosclerosis (Orekhov et al., 2013; Orekhov et al., 2015; Sobenin et al., 2016a). Garlic extracts were demonstrated to impede sialidase activity in blood plasma, which causes atherogenic LDL formation (Aksenov et al., 2007; Venkataiah et al., 2016; Sobenin et al., 2017). Diallyl disulfide, a component of garlic oil, is able to inhibit 3-hydroxy-3-methylglutaryl CoA reductase (HMGR) activity due to significant decrease in the mRNA levels and protein expression of HMGR. (Rai et al., 2009). It was shown in rat models that garlic consumption led to down-regulation of genes related to lipid metabolism: acetyl-CoA carboxylase (ACC), acyl-CoA cholesterol acyltransferase (ACAT), HMGR, fatty acid synthase (FAS), sterol regulatory element-binding protein-1c (SREBP-1c), and glucose-6-phosphate dehydrogenase (G6PD) (Ha et al., 2015). Thus, garlic can cause the lowering of total cholesterol and triglycerides in the blood. It was reported in review of Shouk et al., that bioactive components of garlic possess antihypertensive activity through modulating various parameters implicated in the pathogenesis of hypertension, in particular, proliferation of vascular smooth muscle cells, nitric oxide bioavailability, hydrogen sulfide production, angiotensin converting enzyme activity, expression of nuclear factor-κB (Shouk et al., 2014).

Phytoestrogens, plant-derived estrogens, can be used as an alternative estrogen replacement therapy for Cardiovascular disease (CVD) and osteoporosis prevention. Phytoestrogens, bioflavonoids characterized by similar structure to endogenous estrogens, can be found in different plants like soybean, pomegranate, spinach, etc (Franke et al., 1998; Kirichenko et al., 2017). Soybean contents a lot of isoflavones, which are selective estrogen receptor modulators (Franke et al., 1998; Oseni et al., 2008). Soybean consumption leads to the suppression of VCAM-1 expression (Kim et al., 2019) and elevation of the number of endothelial progenitor cells thus improving endothelial function (Chan et al., 2011). The flavanone 8-prenylnaringenin from hop flowers possessed anti-aggregatory and anti-adhesive effects on human platelets, acting as an inhibitor of platelet aggregation stimulated by different agonists and platelet adhesion to collagen matrix, a direct activator of intracellular cAMP and cGMP production, and VASP phosphorylation promoter (Di Vito et al., 2012). Grape phytoestrogens consumption helps to reduce serum atherogenicity, namely, intracellular cholesterol accumulation in primary culture of human blood-derived monocytes (Nikitina et al., 2006).

In traditional Chinese medicine plants and their mixtures are actively used to treat cardiovascular diseases, including atherosclerosis. Recently researches on the functions of several popular Chinese plants were conducted but they didn't reveal the exact mechanism of action for many of these plants (Hao et al., 2015; Hao et al., 2017; Sedighi et al., 2017). In overall, Chinese plants may influence all pathogenic mechanisms underlying atherosclerosis, including lowering plasma LDL-cholesterol, reduction of oxidative stress in endothelium, attenuation of endothelial proinflammatory activation, inhibition of endothelial cells apoptosis, anti-inflammatory activity, activation of macrophages M2 polarization, decrease of foam cells formation, inhibition of platelet activation and aggregation. Most important Chinese plants are turmeric (*Curcuma longa* L.), Chinese sage or danshen (*Salvia miltiorrhiza* Bge), flowering plants of the barberry family (*Berberidaceae family* Juss.), green chireta (*Andrographis paniculate* Nees.), oak leaves (*Quercus dentata* Thunb.), garlic (*Allium sativum* L.), and jiaogulan (*Gynostemma pentaphylla* Thunb.) (Qiao and Chen, 2018). For example, curcuma may reduce cholesterol accumulation in arterial wall by inhibiting SR-A-mediated oxidized LDL uptake and promoting cholesterol efflux through activation of AMPK-SIRT1-LXRα signaling (Zhao et al., 2012; Lin et al., 2015). Curcumin, which is an active component of turmeric, exhibits its anti-inflammatory properties *via* negative regulation of pro-inflammatory interleukins (IL-1, IL-2, IL-6, IL-8, and IL-12), cytokines (TNF-a), MCP-1) by inhibiting expression of JAK/STAT signaling pathway. Moreover, curcumin may regulate the inflammatory response by suppressing activity of iNOS, COX-2, lipoxygenase, and xanthine oxidase, and activation of NF-kB (Kocaadam and Şanlier, 2017).

Chinese sage has a potential to inhibit foam cell formation caused by oxidized LDL through down-regulating of CD36 expression and up-regulating of Prdx1/ABCA1 signaling (Bao et al., 2012; Yang et al., 2018). Tanshiones, a group of lipophilic abietane diterpene compounds from Salvia miltiorrhiza, stabilized plaques or even attenuated plaque formation through inhibition of NF-kB pathway (Liu et al., 2015; Yang et al., 2016; Zhao D. et al., 2016; Zhao W. et al., 2016), suppressed endothelial apoptosis *via* lncRNA TUG1 up-regulating miR-26a expression (Chen et al., 2016). Tanshiones also protect from oxidation stress by increasing NO production and superoxide dismutase activity, inhibiting ROS production (Lin et al., 2006; Yang et al., 2011; Chen et al., 2012; Joe et al., 2012). Salvia compounds possess anti-thrombotic activity since they inhibit phosphoinositide 3-kinase and that's why decrease platelet activation (Huang et al., 2010). Chinese sage and gegen (*Pueraria lobate* Willd.) radix is a herbal formula suppress the expression of ICAM-1 and VCAM-1 and production of MCP-1 in TNF-α stimulated human umbilical vein endothelial cells (Koon et al., 2011). Sage also has antihypertensive activity as it was shown that sage extract increased nitric oxide production and relaxed endothelium-intact rings in experiments on isolated thoracic aorta from rats (Anwar et al., 2017).

Berberine from Huang Liang (*Coptis chinensis* Franch.) decreased plasma LDL-cholesterol by upregulating LDLR, and apoE expression and downregulating HMGR expression (Kong et al., 2004; Chang et al., 2012). Berberine exhibits cytoprotective effect on endothelial cells (EC) by blocking JNK phosphorylation (Guo et al., 2016) and increases plaque stability by inhibiting MMP-9 and EMMPRIN by suppressing the activation of p38 pathway (Huang et al., 2011) and NF-kB activation (Huang et al., 2012). Foam cell formation can be reduced by berberine *via* enhancing LXRα-ABCA1-dependent cholesterol efflux (Lee et al., 2010). Berberine activates the AMPK-SIRT1-PPAR-γ pathway and, respectively, reduces the uptake of oxidized LDL (Chi et al., 2014). Coptis chinensis extract can induce macrophage autophagy *via* AMPK/mTOR and PI3K/AKT/mTOR pathways (Fan et al., 2015; Kou et al., 2017). More Chinese herbs, their influence on atherosclerosis and underlying mechanism were thoroughly described in recent reports (Zhao et al., 2012; Hao et al., 2015; Qiao and Chen, 2018).

Mulberry (*Morus alba* L.) leaf extract provides anti-platelet and antithrombotic effects *via* blocking of platelet activation and aggregation, thromboxane B2 formation, serotonin secretion, and thrombus formation. Granule secretion and extracellular-signal-regulated kinase and Akt phosphorylation underlie the mechanism of this anti-platelet activity (Kim et al., 2014). Mulberry leaf extract protects lymphocyte DNA from oxidative damage, prevents ROS-mediated endothelial cell dysfunction *via* downregulating intracellular redox-dependent signaling pathways. It also has anti-inflammatory effects through the modulation of AP-1, NF-κB, PPARs, and STAT3 signaling (Chao et al., 2013).

The last but not the least medicine plant in this review is pomegranate (*Punica granatum* L.), which extracts possess multiple anti-atherosclerotic activities. Thus, ellagic acid promotes cholesterol removal by regulating LXR/PPAR-ABCA1 pathway (Zhao S. et al., 2016). Pomegranate peel extract and punicalgin up-regulate mRNA expression of LXRα and ABCA1 (Zhao et al., 2014). Ellagic acid and punicalagin cause anti-oxidant effect by reducing ROS level produced by mitochondria (Hwang et al., 2010; Zou et al., 2014). Moreover, pomegranate possesses obvious anti-inflammatory effect since pomegranate peel, flower, and seed oil may reduce plasma levels of IL-6 and TNFa, pomegranate flower may increase the anti-inflammatory cytokine IL-10 (Harzallah et al., 2016) and, in addition, pomegranate extract diminishes the translocation of NF-κB from the cytosol to the nucleus (Mandal et al., 2017). Pomegranate ellagic acid, peel polyphenols, and punicalagin lower LPS-induced pro-inflammatory cytokine activation (Du et al., 2018).

The most common pathogenetic mechanisms of anti-atherosclerotic action of medicinal plants are presented in **Table 1**.

**Table 1:** Possible Anti-Atherosclerotic Mechanisms of Action of Medicinal Plants.

Effect	Plants/complex of plants	Mechanism
Anti-inflammatory (suppression of inflammatory mediators expression and signal pathway)	1) Inflaminat (Calendula *(Calendula officinalis* L.) + Black Elder (*Sambucus nigra* L.) + Violet (*Viola tricolor* L.))	Inhibiting expression of IL-1β and TNF (Kirichenko et al., 2016)
	Licorice (*Glycyrrhiza glabra* L.)	- JNK and NF-κB signaling blocking (Kwon et al., 2007; Cook-Mills et al., 2011) - TNF-α, IL-1β production suppression (Park et al., 2010) - Adhesion molecules suppression (Jong et al., 2006; Chang et al., 2010)
	Garlic (*Allium sativum* L.)	- Inflammation signaling inhibiting (like TNF-α, IL-1β, ICAM-1 and HLA-DR) (Karagodin et al., 2016a)
	Turmeric (*Curcuma longa* L.)	- Inhibiting expression of JAK/STAT signaling pathway (Kocaadam and Şanlier, 2017) - Suppressing activity of iNOS, COX-2, lipoxygenase, and xanthine oxidase (Kocaadam and Şanlier, 2017) - Activation of NF-kB (Kocaadam and Şanlier, 2017)
	Chinese sage (*Salvia miltiorrhiza*)	- Inhibition of NF-kB pathway (Yang et al., 2016; Zhao D. et al., 2016)
	Chinese sage (*Salvia miltiorrhiza*) and gegen (*Pueraria lobate* Willd.)	- Inhibition of adhesion molecules expression (Koon et al., 2011) - Suppression of MCP-1secretion (Koon et al., 2011)
	Huang Liang (*Coptis chinensis* Franch.)	- JNK phosphorylation (Guo et al., 2016) - Activation of p38 pathway (Huang et al., 2011) - NF-kB activation (Huang et al., 2012)
	Mulberry *(Morus alba* L.)	- Modulation of AP-1, NF-κB, PPARs, and STAT3 signaling (Kim et al., 2014)
	Pomegranate (*Punica granatum* L.)	- Reduction plasma levels of IL-6 and TNFa and increase IL-10 (Harzallah et al., 2016)
Antioxidant (Inhibition of LDL oxidation)	Licorice extract (*Glycyrrhiza glabra* L.)	- Inhibiting of LDL oxidation by suppression of AAPH-induced formation of cholesteryl linoleate hydroperoxide in LDL particle (Rosenblat et al., 1999; Kang et al., 2015)
	Chinese sage (*Salvia miltiorrhiza* Bge.)	- Inhibiting ROS production (Lin et al., 2006; Huang et al., 2010; Yang et al., 2011; Joe et al., 2012)
	Mulberry *(Morus alba* L.)	- Down-regulating intracellular redox-dependent signaling pathways (Chao et al., 2013)
	Pomegranate (*Punica granatum* L.)	- Reducing ROS level production by mitochondria (Harzallah et al., 2016; Mandal et al., 2017)
Antithrombotic activity (prevention of platelet aggregation)	Garlic (*Allium sativum* L.)	- Suppression of cyclooxygenase-2 (Ali et al., 2000) - Reducing synthesis of vasoconstrictors such as prostaglandin E2 and leukotriene C4 (Vilahur and Badimon, 2013) - Regulation of serotonin and coagulation factor IV production (Rahman, 2007) - Supression of platelet aggregation by increasing of cyclic nucleotides (Allison et al., 2012; Rahman et al., 2016) - Inhibiting the GPIIb/IIIa receptor and fibrinogen binding (Morihara and Hino, 2017) - Suppressing the phosphorylation of collagen-induced ERK, JNK and p38 (Morihara and Hino, 2017)
	Hop flowers (*Hunulus lupulus* L.)	- Increasing of intracellular cAMP and cGMP lexpression, promotion of VASP phosphorylation (Di Vito et al., 2012)
	Chinese sage (*Salvia miltiorrhiza* Bge.)	- Inhibition of phosphoinositide 3-kinase (Huang et al., 2010)
	Mulberry *(Morus alba* L.)	- Blocking of platelet activation and aggregation, thromboxane B2 formation (Kim et al., 2014)
Anti-atherogenic (suppression of Intracellular cholesterol accumulation in cultured cells)	Garlic (*Allium sativum* L.)	- Down-regulation of HMGR, FAS, SREBP-1c, G6PDH, acetyl-CoA carboxylase, and ACAT (Rai et al., 2009) - Inhibiting HMGR activity (Venkataiah et al., 2016)
	Turmeric (*Curcuma longa* L.)	- Inhibiting of SR-A-mediated oxidized LDL uptake (Zhao et al., 2012; Lin et al., 2015) - Promoting ABCA1-dependent cholesterol efflux through activation of AMPK-SIRT1-LXRα signaling (Zhao et al., 2012; Lin et al., 2015)
	Chinese sage (*Salvia miltiorrhiza* Bge.)	- Down-regulating of CD36 expression (Bao et al., 2012; Yang et al., 2018) - Up-regulating of Prdx1/ABCA1 signaling (Bao et al., 2012; Yang et al., 2018)
	Huang Liang (*Coptis chinensis* Franch.)	- Up-regulating of LDLR (Kong et al., 2004; Chang et al., 2012) - Up-regulating apoE expression (Kong et al., 2004; Chang et al., 2012) - Down-regulating HMGR expression (Kong et al., 2004; Chang et al., 2012) - Enhancing LXRα-ABCA1-dependent cholesterol efflux (Lee et al., 2010) - Activating the AMPK-SIRT1-PPAR-γ pathway (Chi et al., 2014)
	Pomegranate (*Punica granatum* L.)	- Regulating LXR/PPAR-ABCA1 pathway (Zhao S. et al., 2016) - Up-regulation of LXRα and ABCA1 expression (Zhao et al., 2014)
Hypotensive effects	Garlic (*Allium sativum* L.)	- Inhibition of angiotensin-converting enzyme (Asdaq and Inamdar, 2010) - Activation of the growth suppressor p27 (Castro et al., 2010) - Down-regulation of angiotensin II receptor (Mansour et al., 2013) - Reduction of vasoconstrictor prostanoids synthesis (Al-Qattan et al., 2001) - Stimulation of H2S production (Al-Qattan et al., 2006) - Regulation of endothelial NO synthesis (Ried and Fakler, 2014; Karagodin et al., 2016b)

## Reduction of the Atherosclerosis Risk Factors with Medicinal Plants

Conventional risk factors for cardiovascular diseases, in particular, hyperlipidemia, arterial hypertension, type 2 diabetes mellitus (T2D), smoking, obesity, etc., explain more than 50% of the variability of atherosclerosis, so represent the most significant factor in atherosclerosis development. In this regard, cardiovascular risk factors are the most important target in the process of timely prevention and treatment of atherosclerosis.

Medicinal plants have long been widely studied as anti-atherosclerotic agents; to date, sufficient data have been obtained on the effect of natural products on various traditional risk factors (**Table 2**). Nowadays natural products are known to have the greatest potential for long-term prevention of atherosclerosis.

**Table 2:** Medicinal Plants in Cardiovascular Risk Improvement.

Plant/complex of plants	Study design and results	References
Chinese sage (*Salvia miltiorrhiza* Bge.) and Gegen Radix (*Pueraria lobate* Willd.)	12-months randomized, placebo-controlled trial in postmenopausal women with hypercholesterolemia: - lipid-lowering effect (TC -6.2% and LDL - 7.3%)	Koon et al. (2013)
YH1 (*Coptis chinensis* Franch., *Salvia miltiorrhiza* Bge., *Atractylodis macrocephalae* DC., *Glycyrrhiza glabra* L., *Poria* Pers., *Citrus reticulata* Blanco., *Nelumbinis* Adans., *Platycodon* A.DC., *Amomum* Roxb., *Coix lacryma-jobi* L., *Dolichos* L.)	12-week randomized, double-blind, placebo-controlled pilot trial in 46 patients with T2D: - reduction in HbA1c (-11.1%) - postprandial glucose decrease (-26.2%) - lipid-lowering effect (Tg -29.5%, TC -21.6%, LDL -17.4%)	Huang et al. (2019)
JTTZ formula (*Salvia miltiorrhiza* Bge., *Aloe vera* (L.) Burm.f., *Coptis chinensis* Franch., *Anemarrhena asphodeloides* Bge., *Monascus purpureus* Went., 1895, *Momordica charantia* L., *Schisandra chinensis* (Turcz.) Baill., *Zingiber officinale* Roscoe.	12-week randomized, positive-controlled (metformin), open-label trial in 450 patients with T2D, obesity and hyperlipidemia: - reduction of HbA1c (-0.75 ± 1.32%) - lipid-lowering effect (Tg -0.64 ± 2.37 mmol/L) - Weight decrease (-2.47 ± 2.71 kg)	Yu et al., (2018)
Allicor (*Allium sativum* L.)	4-week randomized, double-blind, placebo-controlled trial in 42 men: - lipid-lowering effect (TC -7.6 ± 2.4%, LDL -11.8 ± 4.5%, Tg -7.7 ± 9.0%)	Sobenin et al. (2008a)
	16-weeks randomized, placebo-controlled, double-blind trial in 84 men with moderate hypertension: - hypotensive (SBP -9.3 ± 0.7 mmHg, DBP -3.8 ± 0.5 mmHg)	Sobenin et al. (2009)
	4-weeks randomized double-blinded placebo-controlled outpatient clinical trial of 60 T2D patients: - blood glucose decrease (-1.8 ± 0.5mmol/l)	Sobenin et al. (2008)
Garlic (*Allium sativum* L.)	6-weeks randomized, double-blind, placebo-controlled trial in 51 patients with obesity: - LDL-lowering effect (p=0.05)	Xu et al. (2018)
Raw crushed garlic	4-weeks open-label trial in 40 patients with metabolic syndrome: - blood pressure decrease (p < 0.001) - Tg -lowering effect (p < 0.01) - blood glucose decrease (p < 0.001) - HDL increase (p < 0.001)	Choudhary et al. (2018)
Aged black garlic	12-weeks open-label trial in 60 patients with with mild hypercholesterolemia: - HDL increase (p < 0.001)	Jung et al. (2014)
Galois (garlic powder tablet)	3-months randomized, double-blind, placebo-controlled trial in 56 patients with coronary artery disease: - SBP decrease (p=0.04)	Mahdavi-Roshan et al. (2016)
Phytoestrogens (*Glycine max* (L.) Merr. + *Passiflora* L.) with vitamins	6-months pilot randomized trial in 90 women in menopausal transition: - TC-lowering effect (p < 0.05)	Villa et al. (2017)
Soy nut (*Glycine max* (L.) Merr.)	8-week randomized placebo-controlled trial in 70 patients with T2D - lipid-lowering effect (TC(p < 0.01) and LDL (p=0.01) decrease) - blood glucose decrease ((p=0.03)	Sedaghat et al. (2019)
Karinat (*Vitis vinifera* L., *Camellia sinensis* L., *Hunulus lupulus* L., *Allium sativum* L.)	12-months randomized double-blind placebo-controlled study in 157 postmenopausal women: - lipid-lowering effect (TC -6.3% (p=0.011), LDL -7.6% (p=0.040)	Myasoedova et al. (2016)
Isoflavones from soybean (*Glycine max* (L.) Merr.)	2-year double‐blind randomized study in 200 early postmenopausal women: - SBP-reduction (-3.2 mmHg, p < 0.01)	Sathyapalan et al. (2018)
Curcumin	10-weeks randomized double-blind placebo-controlled trial in 44 T2D patients: - hs-CRP decrease (-2.5 ± 4.3 mg/L) - Tg–lowering (-14.2 ± 30.6 mg/dl)	Adibian et al. (2019)
Curcuminoids	8-weeks 80 hyperlipidemic in T2D patients: - BMI and lipid-lowering effect (Tg, TC and LDL decrease, p < 0.05)	Adab et al. (2019)
Curcumin formulation	3-months randomized double-blind placebo-controlled trial in 100 T2D patients: - blood glucose decrease, -9 ± 16 mg/dL	Panahi et al. (2018)
	12-weeks randomized, placebo-controlled trial in 50 patients with non-alcoholic fatty liver disease - BMI decrease (p < 0.001) - TNF-α reduction (< 0.001)	Saadati et al. (2019)
Licorice root extract	12-months randomized placebo-controlled study in 110 patients with hypercholesterolemia: - lipid-lowering effect (TC -7.8%, LDL -4.9%) - hypotensive (SBP -13 ± 13 mmHg, DBP -8 ± 10 mmHg)	Fogelman et al. (2016)

Chinese medicine has achieved significant results in reducing the risk factors of atherosclerosis. Studies of the traditional Chinese formula Danshen Salvia miltiorrhiza + Gegen Radix puerariae in showed a significant decrease in plasma total cholesterol (TC) and LDL in postmenopausal women with hypercholesterolemia after 12 months of daily intake of 1 g of the drug in a randomized placebo-controlled trial (Koon et al., 2013). A randomized, double-blind, placebo-controlled pilot trial involving patients with type 2 diabetes showed that daily intake of 9 g of YH1-formula (Rhizoma Coptidis (*Coptis chinensis* Franch.) and Shen-Ling-Bai-Zhu-San (complex of *Salvia miltiorrhiza* Bge., *Atractylodis macrocephalae* DC., *Glycyrrhiza glabra* L., *Poria* Pers., *Citrus reticulata* Blanco., *Nelumbinis* Adans., *Platycodon* A.DC., *Amomum* Roxb., *Coix lacryma-jobi* L., *Dolichos* L.) led to a significant reduction of not only HbA1c and glucose levels, but also plasma Tg, TC, LDL levels (Huang et al., 2019). Another study of the JTTZ formula {Chinese sage [*Salvia miltiorrhiza* Bge.], Aloe vera [*Aloe vera* (L.) Burm.f.], Huanglian [*Coptis chinensis* Franch.], Rhizoma Anemarrhenae [*Anemarrhena asphodeloides* Bge.], red yeast [*Monascus purpureus* Went., 1895], Kugua [*Momordica charantia* L.], Wuweizi [*Schisandra chinensis* (Turcz.) Baill.], and dried ginger [*Zingiber officinale* Roscoe.]}, involving patients with type 2 diabetes, not only reduced HbA1c and normalized the lipid profile of blood plasma, but also led to weight loss (Yu et al., 2018).

Garlic powder is characterized by significant effects on many risk factors of atherosclerosis. In a randomized placebo-controlled trial performed by Zeb F. et al., the weight loss of the subjects was demonstrated (Zeb et al., 2018). In this and other studies, administration of garlic powder caused a significant lipid-lowering effect (Higashikawa et al., 2012; Ahmad Alobaidi, 2014; Alali et al., 2017; Xu et al., 2018; Choudhary et al., 2018). Several clinical trials also confirmed hypotensive effect of garlic. In particular, it was shown in the randomized, placebo-controlled, clinical study of Mahdavi-Roshan et al. that 3-months administration of garlic powder pills (equal to 800 mg garlic daily) results in significant reduction of systolic blood pressure by 12.0 mm Hg in patients with mild arterial hypertension in comparison with placebo (Mahdavi-Roshan et al., 2016). A similar hypotensive effect was demonstrated by other randomized placebo-controlled clinical studies (Ashraf et al., 2013; Al Disi et al., 2016). Treatment with 600 mg garlic powder pills (Allicor) led to a significant decrease of both systolic and diastolic blood pressure by 7.0 and 3.8mmHg (95% CI: 2.7–4.8), respectively (Sobenin et al., 2009). Interestingly, Aalami-Harandi R et al. in their randomized, double-blind, placebo-controlled trial at risk for pre-eclampsia involving pregnant women demonstrated that administration of 400 mg garlic daily for 9 weeks resulted in reduced levels of serum high sensitivity C-reactive protein (hs-CRP) (-1425.90 versus 1360.50 ng/ml, p = 0.01) in comparison with placebo (Aalami-Harandi et al., 2015).

Phytoestrogens are characterized by lipid-lowering and hypotensive effects. Villa P et al. in their pilot randomized trial involving ninety women in menopausal transition showed that administration of phytoestrogen substances in addition to vitamins and passionflower herbal preparation during 6 months led to TC decrease in blood plasma (Saadati et al., 2019). Eight-week of soy nut diet helped to decrease of fasting blood glucose as well as to improve blood lipids profile, significantly decreasing TC and LDL (Fogelman et al., 2016). Other 6-month randomized controlled trial showed the LDL and hs-CRP reduction effects of daily 40 g phytoestrogen-rich soy flour treatment of equol-producing postmenopausal women (Zeb et al., 2018). Other randomized controlled study has shown the hypotensive effect of isoflavones manifesting in a significant decrease of blood pressure (Alali et al., 2017). In a double-blind, randomized, placebo-controlled trial, 50 mg of isoflavone-rich preparation Rimostil resulted in significant serum LDL cholesterol reduction in perimenopausal women (Clifton-Bligh et al., 2015). Plant-derived flavonoids are well-documented to play important vasculoprotective role and it was demonstrated in several randomized controlled trials that catechins and quercetin have significant blood pressure lowering effect (Maaliki et al., 2019).

Turmeric also has lipid, hs-CRP, BMI, and glucose lowering effects observing during treatment of type 2 diabetes. Curcumin, a natural polyphenol from turmeric was tested in a randomized, double-blind, placebo-controlled trial on patients with diabetes who consumed 1,5 g curcumin daily for 10 weeks. Adibian M et al. have found significant hs-CRP and Tg-lowering effects (Adibian et al., 2019). Adab Z et al. have demonstrated that administration of turmeric (2,1 g powdered rhizome of turmeric daily for 8 weeks) significantly decreased BMI as well as plasma Tg, TC, and LDL (Adab et al., 2019). Panahi Y et al. also observed lipid-lowering effect in their randomized controlled trial using curcuminoids (1 g/day plus piperine 10mg/day) treatment of diabetes patients for 12 weeks (Panahi et al., 2018). Finally, a treatment with amorphous dispersion curcumin formulation (500 mg/day equivalent to 70-mg curcumin) of non-alcoholic fatty liver patients for 8 weeks resulted in BMI-reduction as well as plasma Tg, LDL, and glucose levels lowering (Saadati et al., 2019).

In the placebo-controlled clinical study of the effect of 1-year licorice root extract consumption on cardiovascular risk factors, beneficial results were demonstrated, namely, the significant reduction of TC and LDL levels as well as hypotensive activity (Fogelman et al., 2016).

## Potential Direct Anti-atherosclerotic Efficacy of Medicinal Plants

The intima-media thickness of common carotid arteries is often used as a direct quantitative characteristic of atherosclerosis in clinical studies of the effectiveness of anti-atherosclerotic preparations. Currently, a number of medicinal plants have been studied using ultrasound scanning of the carotid arteries, that allows evaluating not only the indirect anti-atherosclerotic effect of these natural products by conversion of cardiovascular risk factors, but also studying their effect on carotid atherosclerosis development. **Table 3** demonstrates the results of clinical trials evaluating the effect of medicinal plants on carotid IMT dynamics during long-term administration.

**Table 3:** Carotid IMT in Studies of the Direct Anti-atherosclerotic Effect of Medicinal Plants.

Plants/complex of plants	Carotid IMT dynamics	References
Chinese herbal formula (danshen *Salvia miltiorrhiza* Bge., gegen radix *Pueraria lobate* Willd.)	12-months cIMT reduction: –0.012 mm (p < 0.001)	Woo et al. (2013); Kwok et al. (2014)
Chinese herb extraction (Reynoutria japonica rhizoma *Polygonum cuspidatum* Siebold & Zucc., hawthorn fruits *Crataegus* Tourn.ex L.)	6-months cIMT reduction (p < 0.05)	Liu et al. (2014)
Allicor (garlic powder *Allium sativum* L.)	annual cIMT change at 2-year study: Allicor –0.022 vs. placebo +0.015 mm/year (p < 0.05)	Karagodin et al. (2016a) (clinical trial registration number NCT01734707, http://clinicaltrials.gov/)
Garlic powder tablets (*Allium sativum* L.)	3-months cIMT dynamics: Garlic –0.009 mm vs. placebo +0.004 mm	Mahdavi-Roshan et al. (2013) (clinical trial registration number NCT01948453, http://clinicaltrials.gov/)
Karinat (*Vitis vinifera* L., *Camellia sinensis* L., *Hunulus lupulus* L., *Allium sativum* L.)	12-month cIMT progression: Karinat +0.006 mm vs. placebo +0.011 mm	Myasoedova et al. (2016) (clinical trial registration number NCT01742000, http://clinicaltrials.gov/)
Soybean (*Glycine max* (L.) Merr.) - isoflavone soy protein	annual cIMT change at 2-year study: ISP +0.002 vs. placebo +0.006 mm/year (p=0.05)	Hodis et al. (2011)
Inflaminat (Calendula flowers *Calendula officinalis* L., Black elder berries *Sambucus nigra* L., Violet herb *Viola tricolor* L.)	annual cIMT change at 2-year study: Inflaminat +0.006 vs. placebo +0.022 mm/year (p=0.045)	Kirichenko et al. (2016) (clinical trial registration number NCT01743404, http://clinicaltrials.gov/)
Licorice root extract (*Glycyrrhiza glabra* L.)	3-months cIMT dynamics: Licorice –0.008 mm vs. placebo +0.003 mm	Fogelman et al. (2016)
Pomegranate (*Punica granatum* L.)		Davidson et al. (2009) (clinical trial registration number NCT00728299, http://clinicaltrials.gov/)

Several medicinal plants that are widely used in Chinese traditional medicine have been studied in clinical trials in patients with carotid atherosclerosis. Chinese herbal formula—complex of Chinese sage (*Salvia miltiorrhiza* Bge.) and gegen radix (*Pueraria lobate* Willd.), that is often used for cardiovascular protection, caused the reduction of cIMT by –0.012mm after 1-year administration in 165 postmenopausal women (Kwok et al., 2014). Similar results of this herbal preparation (D&G) were obtained in a parallel study in high-risk hypertensive patients (Woo et al., 2013). Clinical study of the other Chinese complex: Reynoutria japonica rhizoma (*Polygonum cuspidatum* Siebold & Zucc.) and hawthorn fruits (*Crataegus*Tourn.ex L.) also demonstrates a significant reduction of cIMT after 6 months of follow-up (Liu et al., 2014).

Among a number of medicinal plants that have been studied in terms of anti-atherosclerotic mechanisms of action, garlic seems to be one of the most studied, its effectiveness is also investigated in clinical trials evaluating its direct anti-atherosclerotic action on carotid atherosclerosis. For the first time in 1999 the effect of 4-year garlic powder dragees administration on atherosclerosis plaque dynamics was evaluated by B-mode ultrasound of carotid and femoral arteries and the significant reduction of plaque volume increase during follow-up period in garlic-receiving group in comparison with placebo was shown (Koscielny et al., 1999). Atherosclerosis Monitoring and Atherogenicity Reduction (AMAR) study was designed to evaluate effect of time-released garlic powder pills Allicor on dynamics of carotid IMT. It was shown in 2-year double-blinded placebo-controlled trial that Allicor administration leads to cIMT reduction by –0.022mm per year while in placebo group the progression of cIMT was observed with mean annual rate +0.015mm (p < 0.001) (Orekhov et al., 2013; Karagodin et al., 2016b). Another study of garlic powder tablets efficacy on carotid atherosclerosis showed the similar beneficial data, cIMT dynamics differed significantly between verum and placebo groups (p < 0.001): 3-month cIMT reduction –0.009mm in garlic-administrated group vs. 3-months cIMT progression +0.004mm in placebo-administrated group; only preliminary results of this study were published for this moment (Mahdavi-Roshan et al., 2013).

An important area of research is the correction of atherosclerotic changes in postmenopausal women. It is known that women of childbearing age are less likely to suffer from atherosclerosis than men of this age. However, after menopause, this situation evens out. There is evidence of the ability of preparations containing isoflavonoids (phytoestrogens) to inhibit the formation of new atherosclerotic lesions and slow down the development of existing ones. The data of some studies (Myasoedova et al., 2016; Sobenin et al., 2016b) indicate that the use of isoflavonoid-rich herbal preparations, such as Karinat (grape seeds *Vitis vinifera* L., green tea *Camellia sinensis* L., hop cone *Hunulus lupulus* L., garlic *Allium sativum* L.), has therapeutic potential for the prevention of cardiovascular disease in postmenopausal women and in the perimenopausal period. The significant reduction of annual rate of cIMT progression was demonstrated in 2-year double-blinded placebo-controlled study in postmenopausal women – 0.006 vs. 0.011 mm/year in placebo group (p < 0.001) (Myasoedova et al., 2016). Soybean [*Glycine max* (L.) Merr.] is one of the richest sources of phytoestrogens so it's widely used for therapy of climacteric syndrome and it's also successfully used for cardiovascular protection in women after menopause (Sathyapalan et al., 2018). In the study of Hodis HN et al. the annual rate of cIMT progression differed significantly in the group with isoflavone soy protein (ISP) supplementation in comparison with placebo and was +0.002 and +0.006 mm/year, respectively, (p=0.05); but such effect was observed in subgroup of women within 5 years of menopause, and in the total group of men and women the difference in cIMT progression between ISP and placebo control was not significant (Hodis et al., 2011). It was shown in another study that higher habitual soy food consumption is associated with decreased cIMT in middle-aged Chinese adults, wherein a significant interaction between sex and soy food intake on cIMT was observed (p=0.008) (Zhang et al., 2008).

Several studies of the herbal preparation Inflaminat that suppresses inflammatory cytokines expression as well as inhibits intracellular cholesterol accumulation in *ex vivo* models as was described above, has been conducted. Based on them, an optimal dosage regimen of the preparation was developed to achieve a stable anti-inflammatory effect. In patients with subclinical atherosclerosis, a clinical study was designed to evaluate anti-atherosclerotic properties of Inflaminat. As a criterion for assessing the development of atherosclerotic lesions was also used the cIMT indicator. As it turned out, Inflaminat possesses direct anti-atherosclerotic activity since significantly reduces of the cIMT progression during 2-years of administration. It was demonstrated that annual rate of cIMT progression was +0.006 mm/year in Inflaminat-treated group and +0.022 mm/year in placebo group (p=0.045) (Kirichenko et al., 2016). Among other medicinal plants that possess multiple anti-atherosclerotic effects in atherosclerosis models, several products have been also studied in clinical trials of direct anti-atherosclerotic activity. In particular, in the clinical study of Fogelman Y. et al., the effect of 1-year licorice-root extract (*Glycyrrhiza glabra* L.) consumption on cIMT dynamics was examined in 94 participants aged 41–80 years. It was demonstrated that cIMT reducted by - 0.008 mm in licorice-treated group and increased by +0.003 mm in placebo group (Fogelman et al., 2016). In clinical trial of Davidson M.H. et al., the pomegranate juice consumption decreased cIMT progression only in patients with dyslipidemia, but not in total group (Davidson et al., 2009).

Based on this evidence in our knowledge prevention and treatment of atherosclerosis with medicinal plants has certain limitations. The most important fact is that natural products have multiple therapeutic effects, and it will be almost impossible to know which exact nature of the mechanism of action has led to a beneficial effect. This is especially true for natural complexes in which plants can potentiate and/or attenuate the effects of each other due to various multiple mechanisms of action. Almost all studies have a sufficient number of limitations, very few of them are registered, so it is difficult to assess the main outcome indicators and adverse reaction. Most researchers report that there are no side effects, but protocol requirements for recording any side effects are usually not given; moreover, possible interactions with prescription preparations practically are not describe. Therefore, given the importance of long-term prevention of atherosclerosis and reduction of cardiovascular risk, it is recommended to use the medicinal plants possessing pleiotropic anti-atherosclerotic effects in experimental studies those have shown atheroprotective activity in registered clinical trials.

## Conclusion

Critical analysis of the current literature, demonstrate that the accumulated knowledge of traditional herbal based medicine and modern approaches to studying the action of medicinal plants open a veil of secrecy over the mechanisms of action and possible ways of creating and using preparations based on medicinal plants. Medicinal plants facilitate the treatment of atherosclerosis using different pathway and various mechanisms of the action, a number of medicinal plants have prospects for the prevention or treatment of atherosclerosis. However, direct anti-atherosclerosis activity of preparations based on garlic powder was studied wider than others natural products, their ameliorating effect on the progression of cIMT was proved in randomized double-blinded placebo-controlled trials, registrated on https://clinicaltrials.gov/ (Hodis et al., 2011; Mahdavi-Roshan et al., 2013; Karagodin et al., 2016a Karagodin et al., 2016b). Garlic preparations have the most interesting and obvious cardio-protective properties since garlic is able to ameliorate the blood lipids profile by inhibiting cholesterol biosynthesis, reducing of LDL, modulate arterial hypertension, and inhibit platelet aggregation (Sobenin et al., 2008a; Sobenin et al., 2008b; Sobenin et al., 2009; Jung et al., 2014; Mahdavi-Roshan et al., 2016; Myasoedova et al., 2016; Villa et al., 2017; Choudhary et al., 2018; Sathyapalan et al., 2018; Panahi et al., 2018; Xu et al., 2018; Sedaghat et al., 2019; Adibian et al., 2019; Adab et al., 2019). In laboratory experiments, garlic extract inhibits proliferative and inflammatory cellular reactions, possesses anti-cytokine properties, has pronounced antioxidant activity, so reduces the oxidation of LDL, activates the hydralase of cholesterol esters and inhibit ACAT, so reduces the content of cholesterol esters in cells. Thus, currently garlic seems to be the most promising plant for atherosclerosis prevention and treatment. Further comprehensive study of the mechanisms of influence of natural products on key links in the chain of development of atherosclerosis is an important and necessary step on the way to the development of new safe and effective preparations based on medicinal plants. But even modern data already indicate the possibility of using many medicinal plants as an additional therapy for patients with atherosclerosis and as a preventive measure for subjects with high cardiovascular risk.

## Author Contributions

AO and GA conceived and designed the review. TK, VS, NN, IS, VT, and P-YL contributed with the bibliographic research. TK, VS, AM, and NN wrote the manuscript. TK and IS provided table design. P-YL, VT, AO, and GA—review final version approval. AO, VT, and GA also contributed with the paper organization.

## Funding

This work was supported by Russian Science Foundation (Grant #19-15-00010). This research was also supported within the framework of the grant provided by CSP Ministry of the Health Russian Federation, and by the IPAC RAS State Targets Project # 0090-2019-0005” for Gjumrakch Aliev. This work partially was also supported by the Russian Academic Excellence Project “5-100” for the Sechenov University, Moscow, Russia (for Gjumrakch Aliev and Vadim V. Tarasov).

## Conflict of Interest

GA was employed by GALLY International Biomedical Research LLC.

The remaining authors declare that the research was conducted in the absence of any commercial or financial relationships that could be construed as a potential conflict of interest.

## Abbreviations

LDL, low-density lipoproteins; HDL, high-density lipoproteins; TC, total cholesterol; Tg, triglycerides; hs-CRP, high-sensitive C-reactive protein; cIMT, intima-media thickness of carotid arteries; VCAM-1, Vascular cell adhesion molecule-1; ICAM-1, Intercellular adhesion molecule-1; TNF, tumor-necrosis factor; IL, interleukin; HLA-DR - human leukocyte antigen - DR isotype; HUVECs, human umbilical vein endothelial cells; NO, nitric oxide; HMG-CoA, 3-hydroxy-3-methyl-glutaryl coenzyme A reductase; SREBP-1c, sterol regulatory element binding protein-1c (); COX-2, cyclooxygenase-2; BMI, body mass index; T2D, type 2 diabetes mellitus; DBP, diastolic blood pressure; SBP, systolic blood pressure; ACAT, Acyl-CoA-cholesterol-acyltransferase); PPAR, peroxisome proliferator-activated receptors.
